# Competition, Drought, Season Length? Disentangling Key Factors for Local Adaptation in Two Mediterranean Annuals across Combined Macroclimatic and Microclimatic Aridity Gradients

**DOI:** 10.1002/ece3.70513

**Published:** 2024-11-10

**Authors:** Florian Gade, Johannes Metz

**Affiliations:** ^1^ Plant Ecology & Nature Conservation Group, Institute of Biology & Chemistry University of Hildesheim Hildesheim Germany

**Keywords:** aridity gradient, *Brachypodium hybridum*, competition, drought stress, *Hedypnois rhagadioloides*, local adaptation, season length, slope exposure aspect

## Abstract

Competition in mesic sites and drought stress combined with short growing seasons in drier sites are key environmental factors along macroclimatic aridity gradients. They impose a triangular trade‐off for local adaptation. However, as experiments have rarely disentangled their effects on plant fitness, uncertainty remained whether mesic populations are indeed better competitors and drier populations better adapted to drought stress and short season length. Aridity differs also at microclimatic scale between north (more mesic) and south (more arid) exposed hill‐slopes. Little is known whether local adaptation occurs among exposures and whether south exposures harbor conspecifics better adapted to drier climates that could provide adaptive reservoirs under climate change. We sampled two Mediterranean annuals (*Brachypodium hybridum, Hedypnois rhagadioloides*) in 15 sites along a macroclimatic aridity gradient (89–926 mm rainfall) on corresponding north and south exposures. In a large greenhouse experiment, we measured their fitness under drought stress, competition, and short vs. long growing seasons. Along the macroclimatic gradient, mesic populations were better competitors under benign conditions. Drier populations performed no better under drought stress per se but coped better with the short growing seasons typical for drier macroclimates. At microclimatic scale, north exposure plants were slightly better competitors in *H. rhagadioloides;* in *B. hybridum*, south exposure plants coped better with drought under short season length. We demonstrate that local adaptation to drier macroclimates is trading‐off with competitive ability under benign conditions and vice‐versa. Drought escape via short life‐cycles was the primary adaptation to drier macroclimates, suggesting that intensified drought stress within the growing season under climate change challenges arid and mesic populations alike. Moreover, the drier microclimates at south exposures exhibited some potential as nearby reservoirs of drier‐adapted genotypes. This potential needs further investigation, yet may assist populations to persist under climate change and lessen the need for long‐distance migration.

## Graphical abstract

Using an large greenhouse experiment, we disentangled three key drivers for local adaptation along natural aridity gradients: competition, drought stress, and short season length. In this context, we are the first to combine a microclimatic and macroclimatic scale. Our results relate to ecological theory and climate change vulnerability.

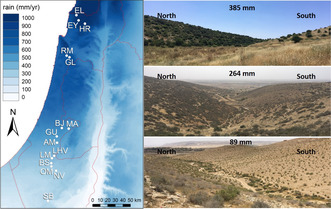

## 1 Introduction

Plant species frequently evolved locally adapted populations along environmental gradients (Kawecki and Ebert 2004; Leimu and Fischer 2008; Hereford 2009; Baughman et al. 2019). However, reviews highlighted that the putative underlying drivers have been rarely tested and disentangled by direct experimental manipulation (Wadgymar et al. 2017, 2022; Johnson et al. 2022). This is further complicated as multiple drivers can act simultaneously and pose trade‐offs along environmental gradients. Regarding which environmental drivers local populations are better adapted and attain higher fitness than other populations has therefore remained uncertain and limits our ability to predict the vulnerability of locally adapted species to climate change (Valladares et al. 2014; DeMarche, Doak, and Morris 2019).

The trade‐off between competitive ability and stress tolerance (Grime 1974, 1977) is widely presumed to govern local adaptation along gradients of increasing aridity such as natural rainfall gradients (e.g., Liancourt and Tielbörger 2009; Kigel et al. 2011; Bergholz et al. 2017; Kurze, Bareither, and Metz 2017; Metz et al. 2020; Galliart et al. 2019, 2020). At the mesic, productive end of aridity gradients, competition is often more intense (Bertness and Callaway 1994; Brooker et al. 2005; He, Bertness, and Altieri 2013; Schiffers and Tielbörger 2006; Liancourt and Tielbörger 2009; Metz and Tielbörger 2016) and should select for increased competitive ability (Grime 1974, 1977). The drier end is characterized by two challenges, overall shorter season length as well as stronger, more frequent drought stress per se (water scarcity) during the growing season due to fewer, smaller, and less predictable rain events (Noy‐Meir 1973; Davidowitz 2002; Drori et al. 2021). Drier sites may therefore select simultaneously for drought escape strategies such as shorter life‐cycles in annuals as well as for increased drought avoidance and tolerance strategies that prevent dehydration (Kooyers 2015).

Abundant common garden studies reported clinal shifts in functional traits along aridity gradients and interpreted them regarding drought stress and competition. For brevity and with respect to our study species, we focus this paragraph on annual species. Populations originating from mesic sites often expressed trait values that indicate increased vegetative growth, e.g. larger vegetative biomass or plant height (e.g., Petrů et al. 2006; Kurze, Bareither, and Metz 2017; Bergholz et al. 2017; Metz et al. 2020; Metz and Tielbörger 2023) or lower reproductive allocation (Aronson et al. 1993; Kurze, Bareither, and Metz 2017; Metz et al. 2020). The authors interpreted these trait values as increased ability to outgrow neighbors under productive, competitive conditions. Drier populations, in turn, were primarily characterized by an earlier onset of reproduction (flowering) and hence shorter life‐cycles (e.g., Del Pozo et al. 2002; Kigel et al. 2011; Kurze, Bareither, and Metz 2017; Ryan and Cleland 2021). Earlier reproduction facilitates escaping intensified drought stress toward the end of the growing season and agrees with the shorter season lengths in drier sites (Kigel et al. 2011; Kooyers 2015). Yet, earlier reproduction may come at the cost of smaller plant size (Kigel et al. 2011; Kurze, Bareither, and Metz 2017) and therefore trade‐off with competitive ability. Moreover, earlier reproduction requires fast growth, causing another trade‐off with drought avoidance especially in drier sites (Wright et al. 2004; Reich 2014; Kooyers 2015). Interestingly, there is thus far little indication that drier populations of annual species reinforced drought avoidance traits that prevent dehydration. Traits such as tougher leaves (lower SLA), increased water use efficiency, and more extensive root systems were rarely stronger (Etterson 2004) and often similar or even weaker in drier populations (Heschel et al. 2004; Bergholz et al. 2017; Kurze, Bareither, and Metz 2017; Metz et al. 2020; Ryan and Cleland 2021). Local adaptation along aridity gradients may therefore pose a triangular trade‐off between competitive ability, stress tolerance, and fast life‐cycles (Grime 1974, 1977).

However, although shifting trait values may be informative indicators, ultimate evidence for local adaptation to competition, drought stress, and short growing seasons requires demonstrating systematic fitness differences among populations (Kawecki and Ebert 2004; Wadgymar et al. 2022). Such direct experimental evidence via plant fitness has remained surprisingly scarce. Some transplant experiments reported patterns of local adaptation along aridity gradients, i.e. local populations tended to outperform foreign populations from other sites (Volis, Mendlinger, and Ward 2002; Etterson 2004; Sambatti and Rice 2006; Liancourt and Tielbörger 2009; Galliart et al. 2019), while other transplants found no such patterns (Ariza and Tielbörger 2011; Tomiolo, van der Putten, and Tielbörger 2015; Galliart et al. 2020). More importantly, demonstrating higher competitive ability of mesic populations (by modifying competition intensity within planting sites) was rarely attempted and more often failed (Sambatti and Rice 2006; Ariza and Tielbörger 2011; Tomiolo, van der Putten, and Tielbörger 2015; Rysavy et al. 2016) than succeeded (Liancourt and Tielbörger 2009). Similarly, several transplant experiments failed to show that drier populations performed best in drier sites (e.g., Liancourt and Tielbörger 2009; Ariza and Tielbörger 2011; Tomiolo, van der Putten, and Tielbörger 2015), and nearly no transplant experiment disentangled in drier sites the effect of short growing seasons from drought stress on fitness (but see Sambatti and Rice 2006). Hence, the role of competition, drought stress, and season length for local adaptation along aridity gradients is insufficiently disentangled and requires stronger direct experimental support than is currently available.

Most above studies investigated macroclimatic aridity gradients across dozens or hundreds of kilometers where geneflow and dispersal between sites are less likely (Siewert and Tielbörger 2010; Giladi, Segoli, and Ungar 2013). However, aridity differs also at much smaller scale. It is well documented for example that south‐exposed hill‐slopes experience higher solar radiation and evapotranspiration than corresponding north exposures (vice‐versa in the southern hemisphere); south exposures therefore provide lower soil moisture, carry lower standing biomass, and harbor higher abundancies of plant species with more arid distribution ranges (Kutiel 1992; Kutiel and Lavee 1999; Finkel, Fragman, and Nevo 2001; Sternberg and Shoshany 2001; Nevo 2012; Gutiérrez‐Jurado et al. 2013; Yang, El‐Kassaby, and Guan 2020; Blanco‐Sánchez et al. 2022). South exposures thus mimic within a few hundred meters major aspects of macroclimatic drier sites (Estevo et al. 2022; Nevo 2012). This implies the intriguing—yet scarcely investigated—prospect that south exposures may harbor drier‐adapted genotypes that could serve as nearby adaptive reservoirs and reduce the need for long‐distance migration under climate change. There have been reports from single sites that plants from south and north exposures diverged genetically or in some functional traits (Nevo 2012; Qian et al. 2018; Wang et al. 2023) when plants were grown under common conditions. Similarly, one study across six sites reported that some traits diverged between conspecifics from north and south exposures, yet often incoherently among sites (Kurze, Bareither, and Metz 2017). Whether such genetic and trait divergence mirrors local adaptation along macroclimatic gradients and translates into higher competitive ability for plants from north exposures and higher tolerance to drought and short season length for conspecifics from south exposures awaits testing.

The present experiment disentangled via plant fitness the triangular trade‐off between competition, drought stress, and season length for local adaptation along combined macro‐ and microclimatic aridity gradients. Two annual species were sampled from corresponding north and south exposures in 15 sites that spanned from mesic to arid conditions along a natural rainfall gradient. Plant fitness was measured as reproductive biomass in a greenhouse under competition, drought stress, and control treatments, each time under short and long season length. We hypothesized that populations from mesic sites maintain higher fitness under competition while populations from drier sites maintain higher fitness under drought stress and short season length. We further hypothesized corresponding responses at microclimatic scale, namely that plants from north exposures suffer less from competition while conspecifics from south exposures suffer less from drought and shorter season length, suggesting that south exposures mimic ‘outposts’ of macroclimatic drier populations.

## 2 Materials & Methods

### 2.1 Sampling Sites

**FIGURE 1:**
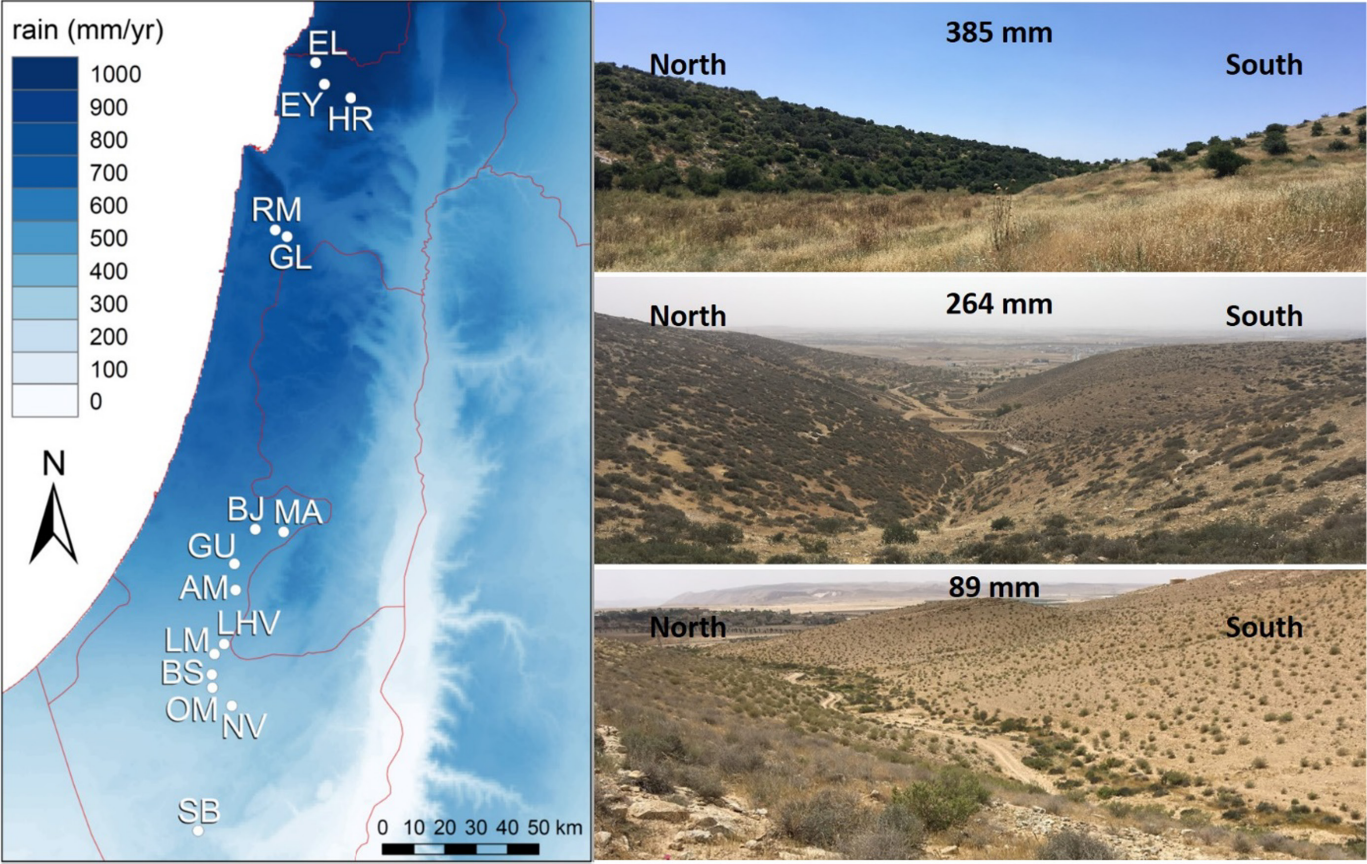
Left: Map of mean annual precipitation in the study region and the location of 15 sampling sites along the macroclimatic rainfall gradient from northern to southern Israel. Right: Illustration of the microclimatic difference between north and south‐exposed hill‐slopes in three exemplary sites. Note the sparser vegetation on south exposures as indication of their drier microclimate. Photo credit: J. Metz.

As macroclimatic aridity gradient, we utilized a steep natural rainfall gradient from northern to southern Israel (Figure 1) that runs parallel to the Mediterranean coast without covarying changes in altitude or temperature (Table 1). We selected 15 sites across 250 km of the gradient that were at least 4 km apart, spanned 89–926 mm mean annual rainfall, and extended from arid to mesic‐Mediterranean conditions (Figure 1, Table 1). The overall climate is Mediterranean with hot, rainless summer seasons and mild, rainy winter seasons. The growing season length is restricted to the period of winter rains, and roughly doubles from the arid end (*c*.3 months, Dec/Jan—March) toward the mesic end (≥ 6 months, Oct/Nov—May) of the gradient. During the growing season, average soil moisture increases (i.e., drought stress per se decreases) roughly threefold from mesic to arid sites (Talmon, Sternberg, and Grünzweig 2011). Net primary productivity and standing biomass of the herbaceous vegetation increases at least six‐ to eightfold from arid to mesic sites (Golodets et al. 2013; Tielbörger et al. 2014), which is reflected in sparse desert vegetation at the arid end and dense Mediterranean woodlands at the mesic end of the gradient (Figure 1, Table 1). Earlier studies showed that competition intensified alongside with productivity toward mesic sites (Schiffers and Tielbörger 2006; Metz and Tielbörger 2016; see also Liancourt and Tielbörger 2009 for a neighboring gradient). Aside from these key differences in rainfall amount, growth season length, and competition intensity, all sites had similar bedrock (limestone) and mean annual temperature (17.8°C–19.8°C) and harbored wood and scrub lands typical for the region (Table 1).

**TABLE 1:** Properties of the 15 sampling sites along the macroclimatic rainfall gradient in Israel.

Site code	Site	Rain (mm/yr)	CV rain (%)	Temperature (°C)	exposure	No. of included genotypes		Altitude a.s.l. (m)	Inclination (°)	GPS location	Vegetation
						*B. hybridum*	*H. rhagadioloides*				
HR	Harashim	926	26	17.8	N	13	15	669	22	32.9646N, 35.3287E	Dense woodland (c.80% tree cover) of Quercus calliprinos, Calicotome villosa, Rhamnus lycioides, Pistacia lentiscus
					S	15	15	750	20	32.9533N, 35.3269E	Open woodland (c. 30% tree cover, 30% shrub cover) of Quercus calliprinos, Calicotome villosa, Pistacia lentiscus
EY	Ein Yaacov	820	26.6	18.9	N	12	12	480	13	33.0057N, 35.2394E	Dense woodland (c.80% tree cover) of Quercus calliprinos, Calicotome villosa
					S	17	16	490	12	33.0067N, 35.2394E	Dense shrubland (c.75% shrub cover) of Calicotome villosa, Sarcopoterium spinosum, Cistus spp., Quercus calliprinos
EL	Eilon	804	25.2	19.8	N	11	3	225	12	33.0688N, 35.2099E	Woodland (c.60% tree cover) of Quercus calliprinos, Calicotome villosa, Sarcopoterium spinosum, Salvia fruticosa
					S	20	16	200	15	33.0697N, 35.2057E	Open shrubland (c. 20% shrub cover) of Sarcopoterium spinosum with single Quercus calliprinos trees (< 10% cover)
RM	Ramot Menashe	666	27.7	19.7	N	19	11	158	15	32.5876N, 35.0613E	Dense grassland (c.70% cover) of annual & perennial herbaceous vegetation, 10% shrub cover of Calicotome villosa
					S	20	7	140	10	32.5885N, 35.0558E	Similar to N‐slope
GL	Gelad	646	26.1	19.5	N	17	15	230	15	32.5677N, 35.1003E	Dense grassland (c.70% cover) of annual & perennial herbaceous vegetation, 10% shrub cover Majorana syriaca, Calicotome villosa, Sarcopoterium spinosum
					S	20	17	230	13	32.5703N, 35.0986E	Similar to N‐slope
MA	Mata	578	29.3	18.1	N	17	9	606	17	31.7117N, 35.0691E	Shrubland (c.70% shrub cover) of Calicotome villosa, Sarcopoterium spinosum, Cistus spp., Quercus calliprinos; herbaceous vegetation between shrubs
					S	20	5	610	17	31.7130N, 35.0665E	Similar to N‐slope, but only c.60% shrub cover
BJ	Bet Jimal	506	30.7	19.8	N	16	16	335	19	31.7212N, 34.9735E	Dense shrubland (c.70% shrub cover) of Cistus spp., Quercus calliprinos, Salvia fruticosa, Pistacia lentiscus, Sarcopoterium spinosum
					S	17	14	335	20	31.7232N, 34.9728E	Shrubland (c.50% shrub cover) of Sarcopoterium spinosum
GU	Bet Guvrin	403	31.4	19.8	N	11	10	302	14	31.6223N, 34.9009E	Shrubland (c.50% shrub cover) of Sarcopoterium spinosum, Pistacia lentiscus, Quercus calliprinos, Rhamnus lycioides
					S	12	7	306	14	31.6238N, 34.9014E	Similar to N‐slope
AM	Amatziya	385	27.5	19.6	N	12	12	329	19	31.5467N, 34.9041E	Shrubland (c.40% shrub cover) of Rhamnus lycioides, Pistacia lentiscus, Sarcopoterium spinosum, Quercus calliprinos

				S	7	15	330	18	31.5480N, 34.9049E	Open shrubland (c.10% shrub cover) of Sarcopoterium spinosum and single trees (Quercus calliprinos, Rhamnus lycioides); dense herbaceous vegetation between shrubs
LHV	Lahav	304	29.5	18.9	N	16	NA	428	17	31.3914N, 34.8622E	Shrubland (c.70% shrub cover) of Sarcopoterium spinosum, Euphorbia hierosolymitana; dense herbaceous vegetation between shrubs
					S	15	12	430	17	31.3925N, 34.8621E	Shrubland (c.40% shrub cover) of Sarcopoterium spinosum, Euphorbia hierosolymitana; herbaceous vegetation between shrubs
LM	Lehavim	264	32.7	19.1	N	15	NA	340	20	31.3624 N, 34.8290° E	Shrubland (50% shrub cover) of Sarcopoterium spinosum; herbaceous vegetation between shrubs
					S	15	12	340	20	31.3636N, 34.8288E	Shrubland (20% shrub cover) of Sarcopoterium spinosum; loose herbaceous vegetation between shrubs
BS	Beer Sheva	242	31	18.9	N	14	15	420	14	31.3037N, 34.8196E	Open shrubland (c.10% shrub cover) of Thymelea hirsuta, Sarcopoterium spinosum; sparse herbaceous vegetation
					S	15	11	420	13	31.3066N, 34.8193E	Open shrubland (c.5% shrub cover) of Thymelea hirsuta, Sarcopoterium spinosum; sparse herbaceous vegetation
OM	Omer	220	29.3	19.2	N	13	14	342	17	31.2653N, 34.8192E	Open shrubland (c.10% shrub cover) of Thymelea hirsuta, Ballota undulata, Teucrium spp.; mainly annuals between shrubs

				S	15	13	340	15	31.2641N, 34.8199E	Open shrubland (c.5% shrub cover) of Thymelea hirsuta, Ballota undulata; mainly annuals between shrubs
NV	Nevatim	138	37.5	19.2	N	15	13	381	15	31.2120N, 34.8840E	Open shrubland (c.25% shrub cover) of Echinops spp.; mainly annuals between shrubs
					S	NA	11	380	15	31.2126N, 34.8840E	Open shrubland (c.10% shrub cover) of Echinops spp.; mainly annuals between shrubs
SB	Sde Boqer	89	42.3	18.8	N	NA	NA	470	15	30.8528N, 34.7647E	Open shrubland (c. 5% shrub cover) with Zygophyllum dumosum, Artemisia sieberi, Hammada scoparia; sparse annual cover between shrubs
					S	14	NA	470	15	30.8537N, 34.7639E	Open shrubland (< 5% shrub cover) with Zygophyllum dumosum, Artemisia sieberi, Hammada scoparia; sparse annual cover between shrubs

*Note:* Site names refer to the nearest town. Mean annual precipitation (rain) for each sampling site was obtained from the nearest station of the Israel Meteorological Service (www.ims.gov.il), calculated across the period 1984–2020. The interannual variation of rainfall (CV rain; coefficient of variation in rainfall among individual years) increased toward drier sites. Mean annual temperature per site was obtained from the worldclim.org data base and showed no trend along the gradient. Rain, CV rain, and temperature describe the macroclimate and were not separately available for the microclimatic difference between north and south exposures. Altitude above sea level (a.s.l.) and inclination (i.e., steepness of the slope) were similar between exposures, while the vegetation was usually sparser at south exposures. The experiment included slightly variable numbers of genotypes per site—exposure combination of two target species *Brachypodium hybridum* and *Hedypnois rhagadioloides* as the species were scarce or even absent (NA) in some combinations.

As microclimatic aridity gradient, we utilized the contrast between corresponding north and south exposures within the 15 sites (Figure 1, Table 1). Both exposures were usually 150–300 m apart, separated by a small valley, and had similar elevation, inclination, and vegetation structure (Figure 1, Table 1). However, south exposures had lower vegetation cover than their corresponding north exposures (Figure 1, Table 1), indicating that south exposures experience drier microclimates and thus approximate at small spatial scale the conditions of drier sites along the macroclimatic gradient.

### 2.2 Study Species

**FIGURE 2:**
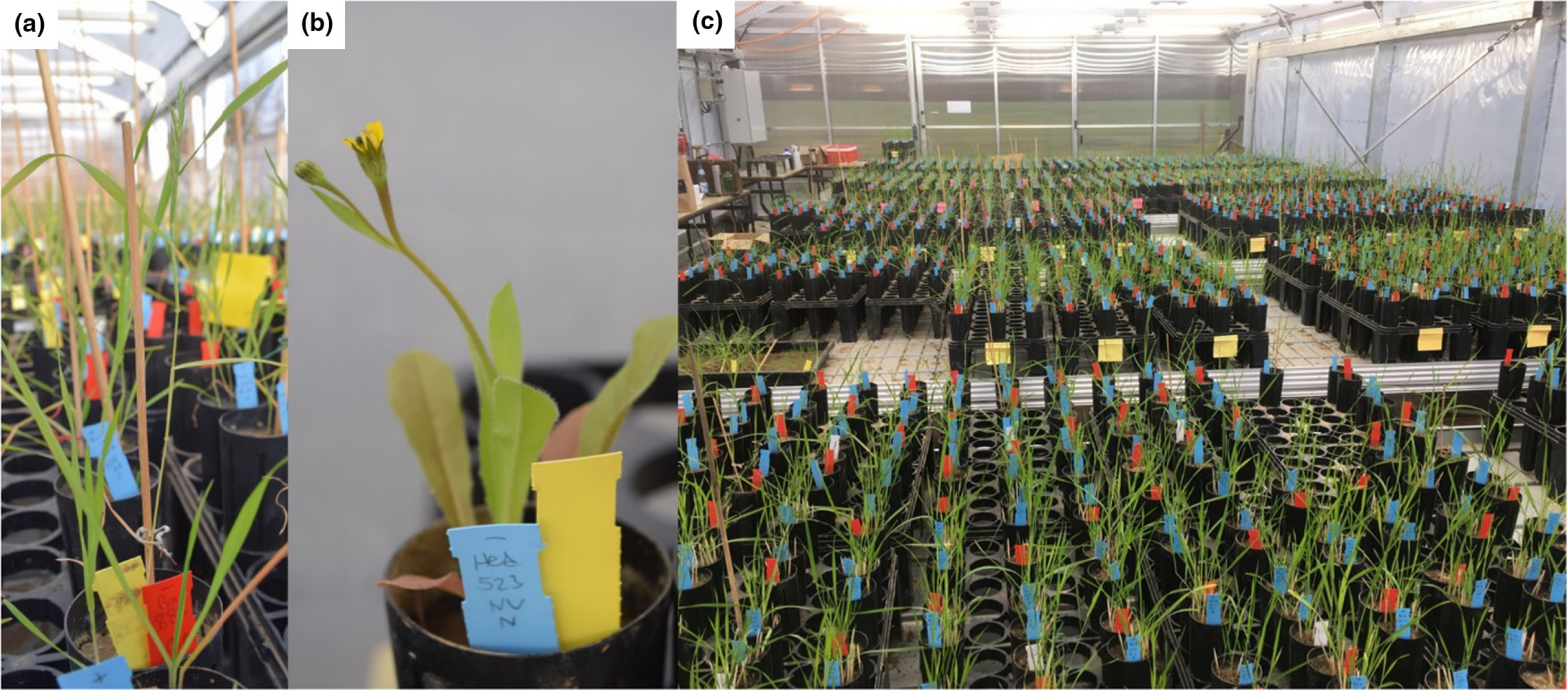
Portraits of the study species *Brachypodium hybridum* (a) and *Hedypnois rhagadioloides* (b) and a view of the greenhouse setup (c). Photo credit: M. Winter (a,c) and F. Gade (b).

We used two annual species, the Poaceae *Brachypodium hybridum* Catalán, Joch. Müll., Hasterok (Figure 2a) & Jenkins and the Asteraceae *Hedypnois rhagadioloides* (L.) F. W. Schmidt (Figure 2b). Both species are common throughout the Mediterranean Basin and cover a wide climatic range from arid to mesic‐Mediterranean conditions (*c*.100 to > 1000 mm annual rainfall) (Feinbrun‐Dothan, Danin, and Plitmann 1998), making them well‐suited to study local adaption. They often co‐occur within the herbaceous layer of shrublands, pastures, and abandoned fields (Feinbrun‐Dothan, Danin, and Plitmann 1998).

*B. hybridum* usually reaches 15–45 cm height (Feinbrun‐Dothan, Danin, and Plitmann 1998) and diverges clearly in functional traits suggestive of local adaptation along our macroclimatic gradient (Kurze, Bareither, and Metz 2017; Penner et al. 2020). *B. hybridum* is pre‐dominantly selfing, allotetraploid (2*n* = 30), and forms together with its diploid ancestors *B. distachyon* (2*n* = 10) and *B. stacei* (2*n* = 20) the complex *B. distachyon* s. l. (Catalán et al. 2012; Hasterok et al. 2022). *B. hybridum* is the dominant species of this complex in our study region, comprising approx. 90% of specimens (Bareither, Scheffel, and Metz 2017). *H. rhagadioloides* (syn. *H. cretica* (L.) Dum.Cours) can reach 40 cm height, is often multi‐stemmed with a variable habit from erect to prostrate, and bears heads with smaller inner and larger outer seeds (Kigel 1992; Feinbrun‐Dothan, Danin, and Plitmann 1998).

### 2.3 Seed Sampling and Plant Cultivation

In spring 2019, seed material was collected in all 15 sites on north and south exposures. If available, seeds of 15 random specimens of both species were collected and bagged separately within a 100 m × 50 m area at each exposure, mostly in the herbaceous layer between shrubs as the species were most abundant there, and keeping at least 3 m distance between conspecific specimens (hereafter: genotypes) for reducing genetic relatedness. At some sites and exposures, however, the species were scarce or absent, resulting in a total of 423 genotypes for *B. hybridum* (mean per exposure 15, range 7–20) and 316 genotypes for *H. rhagadioloides* (mean per exposure 12, range 3–17; detailed numbers in Table 1). All seeds were stored at room temperature in paper bags. From these seeds, we raised one offspring per genotype during the following winter season (Dec 2019—May 2020) in a greenhouse in Hildesheim, Germany, under common conditions and regular watering. In this way, we diminished parental environmental effects from field conditions and amplified seed material. Cross‐pollination was prevented among genotypes to obtain full‐sibling seeds per genotype (as in Metz et al. 2020) and their seeds were bagged separately.

The main experiment was conducted in the next winter season (Nov 2020—May 2021) in the same greenhouse as above. Three pots per genotype were installed that corresponded to three separate treatments (control, competition, and drought; see below), totaling 1269 pots for *B. hybridum* and 948 pots for *H. rhagadioloides*. We used 400 mL pots (Deepot Cells, Stuewe & Sons, Oregon, US: diameter 5.7 cm, depth: 25 cm) filled with silty clay soil from weathered loess near Hildesheim without additional fertilizer (pH 7.2 ± 0.1SD; 2.9% ± 0.1% soil organic carbon; 56 ± 16 ppm total N) that mimicked well the local soils over calcareous bedrock along the large rainfall gradient in Israel (Zwikel, Lavee, and Sarah 2007). Each pot contained five intact seeds of one single genotype. As testing for differences between both species was not our focus, we placed their pots on separate greenhouse tables to ease handling. Within both species, the pots were arranged completely randomly and re‐randomized every 3 weeks to preclude bias from greenhouse position.

The experiment started on Nov 29th 2020 by watering all pots with 40 mL (equaling a 16 mm water column of ‘rainfall’) and further 40 mL on Dec 8th and Dec 20th, to ensure sufficient germination and seedling establishment. After germination had ceased, seedlings were thinned randomly to one target individual per pot on Jan 18th–19th. To approximate field temperatures, daytime greenhouse temperatures ranged 15°C–20°C (minimum 10°C) in winter and increased to 20°C–30°C (max. 35°C) in late spring. For ensuring unbiased phenology, we mirrored the natural course of day length in Israel by operating LED lights for 10 h during the day in December and extended this duration gradually by 15 min each week (Aronson et al. 1992). When *B. hybridum* plants started to flower, they were wrapped in thin, transparent fabric (organza) to prevent pollen dispersal, which was unnecessary for the insect‐pollinated *H. rhagadioloides*.

We applied three treatments—control, drought, and competition—with one pot (i.e., one target plant) per genotype in each treatment. The control treatment provided benign growth conditions and received 40 mL water per pot approximately once per week throughout the experiment. The drought treatment simulated the low water availability in climatically drier sites. Here, pots were watered on the same dates as the control treatment, yet with 10 mL between Dec 29th and Feb 17th and with 15 mL afterwards because the older plants and warmer greenhouse temperatures demanded more water. The drought treatment induced visible signs of wilting, especially before the next watering. The competition treatment simulated the conditions in climatically more mesic sites, i.e. high water availability yet stronger competition. It received identical watering as the control treatment, 40 mL on the same dates as above. Yet, competition pots contained two competitor plants in addition to the target individual: one plant of the annual forb *Plantago afra* L. (Plantaginaceae) and one of the annual grass *Avena sterilis* L. (Poaceae). Both are widely distributed species across Israel and often co‐occur with *B. hybridum* and *H. rhagadioloides*; their seed material originated from intermediate sites (MA, BJ) of the gradient. In each competition pot, six seeds of *P. afra* and three seeds of *A. sterilis* were sown and subsequently thinned to one individual per competitor species after completed germination. When no seedling of *P. afra* or *A. sterilis* emerged (32 pots), we replanted the missing competitor from spare plants that were germinated for this purpose in additional pots under identical conditions.

### 2.4 Fitness Quantification for Short and Long Season Lengths

We quantified the fitness of all target plants for two separate season lengths, in March and in May, corresponding to a short (as in arid sites) and a long season length (as in mesic‐Mediterranean sites), respectively. We used reproductive biomass as fitness measure as it is similar to seed number, yet accounts for potential differences in seed mass among treatments and sampling sites. For the long season length, all target plants were harvested between May 10th—14th 2021 and separated in vegetative (leaves and stems) and reproductive biomass. Reproductive biomass in *B. hybridum* was the summed weight of all spikelets, and in *H. rhagadioloides* it was the summed weight of all heads separated directly below their base. Although most spikelets and heads were fully dry and ripe at harvest, they were dried in unsealed paper bags for another 2 weeks at room temperature (to keep seeds viable) before weighing. In addition, we counted the number of spikelets/heads per individual in order to calculate the average weight of one spikelet/head for each target individual. For the short season length, we estimated reproductive biomass non‐destructively for all target plants. For each *B. hybridum* individual, we counted on March 15th the number of spikelets thus far produced, and similarly on March 20th the number of heads for each *H. rhagadioloides* individual. Half‐developed spikelets/heads were counted as 0.5 to obtain a more gradual measure. These numbers were multiplied with the average weight of one spikelet/head for that specific target individual at harvest (see above) for estimating the reproductive biomass under short season length. We acknowledge that our approach to short season length may exclude putative cues that plants might use in the field to sense ending seasons and adjust their phenology accordingly. However, this seems unlikely because the phenological transition to reproduction (onset of flowering) occurs much before seasons end and responded little to cues such as drought stress in numerous annuals from our study region (Kigel et al. 2011) and both species in our experiment (Winter 2022). We thus expect little bias from our approach to season length.

### 2.5 Data Analyses

Reproductive biomass as our only response variable was analyzed separately for both species with linear mixed models in R 4.1.2 (R Core Team 2021).

First, we tested the response of reproductive biomass (untransformed) to all dimensions of our experiment at once with one full model per species in package *lme4* (Bates et al. 2015). The full model included four predictors and their interactions as fixed effects: *rain* (continuous; the mean annual rainfall of the sampling site along the macroclimatic gradient), *exposure* (categorial; the microclimatic gradient with north vs. south exposures), *treatment* (categorial: control, drought, competition), and *season length* (categorial: short vs. long). Moreover, three nested random factors were included that reflected the nested experimental design: *individual* grouped reproductive biomass for short vs. long season length per target individual; *genotype* grouped the three individuals per genotype (one per treatment); and *site* grouped the genotypes originating from the same sampling site. Significance of the fixed effects was tested via type II Wald F‐tests with Kenward‐Roger approximated degrees of freedom in package *car* (Fox and Weisberg 2019).

Second, because the full models of both species yielded various significant interaction terms (Table A1) that hampered interpretation, we split the data and calculated separate models for short and long season length, again separately for both species and using reproductive biomass as response variable. Accordingly, the split models included *rain*, *exposure*, *treatment* and their interactions as fixed effects and *genotype* nested in *site* as random factors. The split models were calculated in the more flexible package *glmmTMB* (Brooks et al. 2017), which was attempted initially but failed to converge for the full models. Based on residual diagnostics in package *DHARMa* (Hartig 2022), *glmmTMB* allowed us to correct for slightly heterogenous variances (~dispformula) in three of the four split models (*B. hybridum* short season, *H. rhagadioloides* long and short season) and for mild zero inflation (~ziformula) in one split model (*B. hybridum* short season). Significance of fixed effects was assessed with type II Wald chi‐square tests in package *car* (Fox and Weisberg 2019). Posthoc tests were calculated if needed in package *emmeans* (Lenth 2023) using the command ‘emmeans’ for *treatment* factor levels and ‘emtrends’ for differential slopes in *treatment*‐interactions with *rain*.

Because only the full models contained *season length*, we used the full models to interpret *season length* and its interactions, while we focused on the more flexible split models to interpret the remaining effects. However, results for full and split models were congruent (Table 2, Table A1).

**TABLE 2:** Results of linear mixed models testing the response of reproductive biomass to annual rainfall at origin along the macroclimatic gradient (rain), exposure (north vs. south), and treatment (control (c), drought (d), competition (cm)) in two annual species *Brachypodium hybridum* and *Hedypnois rhagadioloides*. Analyses were split for long and short season length. Bold indicates significant *p*‐values (*p* < 0.05).

	*B. hybridum*							
	Long season				Short season			
	df	*χ* ^2^	*p*	Post hoc	df	*χ* ^2^	*p*	Post hoc
Rain	1	0.10	0.7534		1	25.18	**< 0.0001**	
Exposure	1	0.25	0.6171		1	2.18	0.1397	
Treatment	2	3405.54	**< 0.0001**	cm < d < c	2	768.54	**< 0.0001**	cm < d < c
Rain × exposure	1	0.43	0.5120		1	5.77	**0.0163**	
Rain × treatment	2	14.60	**0.0007**	c < cm	2	114.51	**< 0.0001**	c < d < cm
Exposure × treatment	2	4.42	0.1097		2	9.17	**0.0102**	
Rain × exposure × treatment	2	0.24	0.8887		2	6.82	**0.0331**	
*H. rhagadioloides*								
Rain	1	0.10	0.7521		1	24.26	**< 0.0001**	
Exposure	1	0.26	0.6093		1	0.03	0.8565	
Treatment	2	1534.64	**< 0.0001**	cm < d,c	2	747.06	**< 0.0001**	cm < d < cn
Rain × exposure	1	0.55	0.4577		1	2.55	0.1106	
Rain × treatment	2	14.30	**< 0.0001**	c,d < cm	2	72.24	**< 0.0001**	c < d < cm
Exposure × treatment	2	11.71	**0.0029**		2	5.92	0.0519	
Rain × exposure × treatment	2	23.96	**< 0.0001**		2	8.80	**0.0123**	

## 3 Results

Reproductive biomass was recorded for 1244 of the 1269 (98%) individuals of *B. hybridum* and for 614 of the 948 (65%) individuals of *H. rhagadioloides*. The low number for *H. rhagadioloides* was chiefly because germination rates were unexpectedly lower in the experiment (27%) than in preliminary trials (58%; *n* = 50), resulting in 279 empty pots. Another 51 individuals died during the experiment.

**FIGURE 3:**
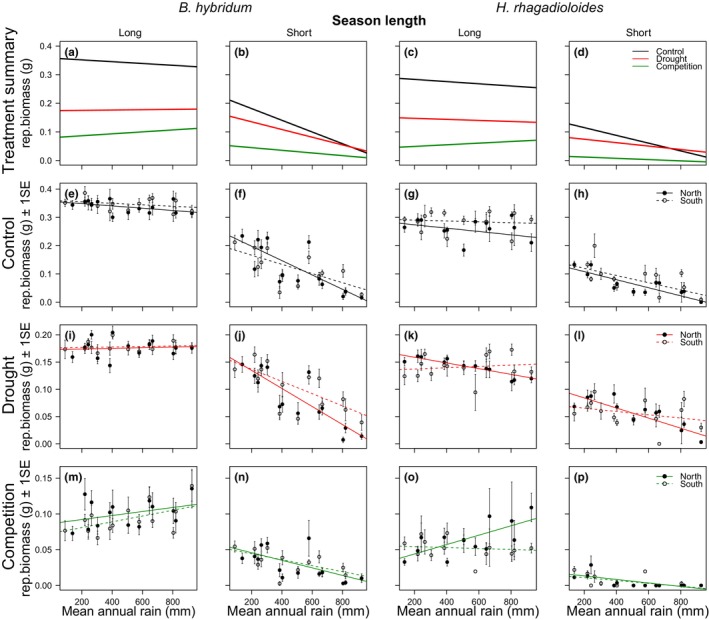
The fitness (quantified as reproductive biomass per plant in all panels) of *Brachypodium hybridum* and *Hedypnois rhagadioloides* populations under combinations of long vs. short season length (vertical columns) and three treatments (control, drought stress, and competition; horizontal rows). To ease comparison, the uppermost panels (a–d) summarize the mean trends per treatment across the macroclimatic rainfall gradient (always on *x*‐axis), i.e. averaged across north and south exposures. The remaining panels (e–p) display the same responses per treatment, yet detailed for north and south exposures and using adjusted *y*‐axes.

Short season length reduced reproductive biomass on average by *c*.50% compared to long season length in both species. Accordingly, *season length* was highly significant in the full models (combining long and short season length) of both species (*B. hybridum p* < 0.0001; *H. rhagadioloides p* < 0.0001; full results in Table A1). Moreover, the drought and the competition treatment reduced reproductive biomass in both species on average by *c*.50% and *c*.70%, respectively, compared to the control treatment (Figure 3a–d). Accordingly, *treatment* was highly significant in both full models (*B. hybridum p* < 0.0001; *H. rhagadioloides p* < 0.0001; Table A1) and all split models (separating long and short season length; Table 2). These significant main effects of *treatment* and *season length* confirmed that our experimental setup was effective in challenging plant fitness. However, central to our hypotheses were the interaction terms of *season length* and of *treatment* with *rain* and *exposure*. They tested whether the season length and treatment effects on reproductive biomass differed with the plants' origin along the macroclimatic (rain) and microclimatic (exposure) gradient. These key interactions are detailed below.

### 3.1 Macroclimatic Gradient

Both species responded in similar ways along the macroclimatic gradient to season length and treatments. In both species, the significant *rain* × *season length* interaction in the full model (*p* < 0.0001 in both species, Table A1) showed that the regression lines with rain inclined (more) negatively under short season (Figure 3b,d) compared to long season length (Figure 3a,c). Hence, the short season length reduced reproductive biomass stronger in mesic than in drier populations in both species.

Also, in both species, the significant *rain × treatment* interaction in all split models (i.e., under both long and short season length; Table 2) indicated that the treatment effects on reproductive biomass changed with origin along the macroclimatic gradient. The post hoc tests in all split models showed that the regression lines with rain were most distinct between the control and the competition treatment (Table 2, Figure 3a–d). In the control treatment, the regression slopes with *rain* were negative in both species and both season lengths (Figure 3a–d,e–h), i.e. drier populations had a fitness advantage (higher reproductive biomass) over mesic populations under control conditions. However, the competition treatment reversed this advantage under long season length (i.e., regression slopes turned positive; Figures 3a,c and 2m,o) and significantly weakened it under short season length (i.e., regression slopes were less negative; Figure 3b,d,n,p). Hence, competition reduced reproductive biomass stronger in drier than in mesic populations. Notably, the drought treatment had always intermediate regression slopes (Table 2, Figure 3a–d), i.e. drought did not enlarge the advantage of drier over mesic populations.

In sum, populations of macroclimatic drier origin suffered less from short season length, stronger from competition, and performed no better under drought than mesic populations in our experiment.

### 3.2 Microclimatic Gradient

In *H. rhagadioloides*, plants originating from north and south exposures responded similarly to long and short season length, as no interaction term was significant in the full model that included *exposure* and *season length* (*exposure × season length, rain × exposure × season length, exposure × season length × treatment, rain × exposure × season length × treatment;* Table A1). Congruently, all terms including *exposure* were either significant in both split models or in none (Table 2). Likewise in *B. hybridum*, plants from north and south exposures were mostly similar under long and short season length, as neither *exposure × season length* in the full model (*p* = 0.45, Table A1) nor the main effect of *exposure* in both split models (Table 2) were significant. However, the weakly significant *rain × exposure × season length* and *exposure × season length × treatment* interactions in the full model (both *p* = 0.025, Table A1) indicated that exposures differed with season length in certain cases that we will detail below by the split models.

In *B. hybridum*, treatments had similar effects on plants originating from north and south exposures under long season length (*exposure × treatment* n.s., Table 2, Figure 3e,i,m). Under short season length, however, the drought treatment slightly favored south exposure plants (*exposure × treatment p* = 0.01, Table 2, Figure 3j). This advantage of south exposure plants under drought was stronger in mesic populations along the macroclimatic gradient and vanished toward arid populations (*rain × exposure × treatment p* = 0.033, Table 2, Figure 3j). In *H. rhagadioloides*, treatment effects differed between north and south exposure plants under long season length (*exposure × treatment p* = 0.0029, Table 2): South exposure plants performed better in the control treatment (Figure 3g) but not better under drought (Figure 3k) while north exposure plants attained higher reproductive biomass in the competition treatment (Figure 3o). This gain under competition was stronger in north exposure plants from mesic populations along the macroclimatic gradient (*rain × exposure × treatment p* < 0.0001, Table 2, Figure 3o). Under short season length, similar patterns emerged but were only marginally (*exposure × treatment p* = 0.052) or weakly significant (*rain × exposure × treatment p* = 0.012, Table 2, Figure 3h,l,p).

In sum, while north and south exposure plants responded overall similarly to long and short season length in both species, the combination of drought and short season length favored south exposure plants in *B. hybridum*, and the combination of long season length and competition favored north exposure plants in *H. rhagadioloides*. Notably, both effects were more pronounced at the mesic end of the macroclimatic gradient.

## 4 Discussion

Our experiment disentangled three key factors—competition, drought stress, short season length—for local adaptation along combined macroclimatic and microclimatic aridity gradients. For both target species, we demonstrated directly via plant fitness that plant populations originating from mesic macroclimates were better competitors under benign conditions, while populations from drier macroclimates coped better with short growing seasons but not better with drought stress per se. Our results align with long‐standing theory proposing a triangular trade‐off between competitive ability, stress tolerance, and fast life‐cycles (Grime 1974, 1977; see also Reich 2014; Kooyers 2015). Moreover, partly corresponding patterns at microclimatic scale suggest that plants from south exposures provide some potential as nearby reservoirs for drier‐adapted genotypes under climate change.

Along the macroclimatic gradient, we found for both species that the fitness (reproductive biomass) of more mesic populations was less suppressed by neighbor competition than the fitness of populations originating from drier sites (Figure 3a–d). This result demonstrated that populations from more benign conditions were better adapted to competition (Grime 1974, 1977). Competition is often stronger in more benign sites along environmental gradients (Bertness and Callaway 1994; Brooker et al. 2005; He, Bertness, and Altieri 2013), including rainier sites along our macroclimatic gradient (Schiffers and Tielbörger 2006; Metz and Tielbörger 2016). Accordingly, many studies on functional traits along aridity gradients argued that populations from more mesic sites may possess better competitive ability because they invested more in traits related to vegetative growth (e.g., Aronson et al. 1993; Kurze, Bareither, and Metz 2017; Galliart et al. 2019, 2020; Metz and Tielbörger 2023). However, direct evidence via plant fitness had remained rare. Although reciprocal transplant experiments along aridity gradients showed that mesic populations often outperformed drier populations in mesic sites (Volis, Mendlinger, and Ward 2002; Etterson 2004; Sambatti and Rice 2006; Liancourt and Tielbörger 2009; Galliart et al. 2019), only one of these studies demonstrated directly via neighbor removal that this fitness advantage resulted from higher competitive ability of the mesic population (Liancourt and Tielbörger 2009). Moreover, several other transplant studies that modified competition intensity found no evidence for mesic populations being better competitors (Sambatti and Rice 2006; Ariza and Tielbörger 2011; Tomiolo, van der Putten, and Tielbörger 2015; Rysavy et al. 2016), and a meta‐analysis indicated little contribution of biotic interactions to local adaptation (Hargreaves et al. 2020). These divergent results suggest that the benefit from investing in competitive traits may not always play out strongly, probably because the intensity of neighbor competition is not constant within natural habitats but varies substantially in space and time (e.g., Kadmon 1995; Seifan, Tielbörger, and Kadmon 2010; Metz and Tielbörger 2016; Rysavy et al. 2016). Transplant experiments, which are often limited in replication, may thus fail to detect differences in competitive ability due to high spatial heterogeneity or unusual weather conditions (Ariza and Tielbörger 2011; Tomiolo, van der Putten, and Tielbörger 2015). Our controlled greenhouse approach avoided such environmental heterogeneity, permitted ample replication, and proved powerful in demonstrating higher competitive ability in more mesic populations.

Another result along the macroclimatic gradient was that drier populations of both species outperformed more mesic populations under short season length in our experiment (Figure 3b,d). Hence, populations originating from drier sites were likely better adapted to shorter growth seasons, which are key features of drier sites along macroclimatic rainfall gradients (Noy‐Meir 1973; Drori et al. 2021). Drier populations flowered earlier in numerous annual species (e.g., Del Pozo et al. 2002; Kigel et al. 2011; Kurze, Bareither, and Metz 2017; Penner et al. 2020; Ryan and Cleland 2021), including both species in the present experiment (Winter 2022). As earlier flowering abbreviates the life‐cycle and permits reproduction under shorter growing seasons (Kigel et al. 2011), it caused most certainly the fitness advantage of drier populations. Interestingly though, we found no indication that drier populations performed better under drought stress per se than more mesic populations (Figure 3a–d). While this finding was surprising at first glance, it aligns with indirect evidence from functional traits in annual species: Traits for withstanding drought stress (dehydration avoidance or tolerance *sensu* Kooyers 2015; Volaire 2018) such as low SLA and extensive root systems were often similar or even weaker in drier populations compared to mesic populations (Heschel et al. 2004; Kurze, Bareither, and Metz 2017; Bergholz et al. 2017; Metz et al. 2020; Ryan and Cleland 2021; but see Etterson 2004). Judging the role of withstanding drought for local adaptation along aridity gradients is not trivial without empirical testing. Although drier sites receive less rainfall, increasing the ability to withstand drought may be curbed in drier populations because it impedes fast growth for drought escape via earlier flowering (Mckay, Richards, and Mitchell‐olds 2003; Reich 2014; Kooyers 2015; Blumenthal et al. 2020). Conversely, in mesic populations, withstanding drought may be favored to survive phases of drought stress during the longer growth season in order to exploit the full season length. Thus far, transplant experiments could not clearly disentangle the coinciding effects of short season length and drought stress per se on plant fitness in drier sites (Volis, Mendlinger, and Ward 2002; Sambatti and Rice 2006; Liancourt and Tielbörger 2009; Galliart et al. 2019). Our experiment showed that adaptation to drier sites in two annual species relied chiefly on escaping drought via shorter life‐cycles and that drier populations were equally susceptible to drought stress as mesic populations.

At microclimatic scale, plants from north and south exposures differed in fitness under certain experimental conditions, suggesting a degree of microclimatic local adaptation. In *B. hybridum*, south exposure plants performed better when drought and short season length were combined (Figure 3j) while in *H. rhagadioloides*, north exposure plants coped better with competition, namely under long season length (Figure 3o). Both effects emerged mostly in macroclimatic more mesic sites, perhaps because soil moisture benefits from lower evaporation on north exposures may persist longer in our mesic sites due to deeper‐developed soils. The different microclimates and plant communities at north and south‐exposed hill‐slopes are well documented (e.g., Kutiel and Lavee 1999; Sternberg and Shoshany 2001; Gutiérrez‐Jurado et al. 2013; Yang, El‐Kassaby, and Guan 2020) and have been proposed as potential genetic reservoirs for climate change adaptation (Nevo 2012; Denney et al. 2020). Yet, tests whether conspecifics from north and south exposures possess adaptations to different microclimates are rare. Indirect support comes from diverging functional traits and genetic composition between plants originating from north and south exposure in single sites in mesic Israel (> 600 mm annual rainfall) (Nevo 2012; Qian et al. 2018; Wang et al. 2023). Moreover, some functional traits diverged between exposures across six of our study sites in *B. hybridum*, yet often inconsistently among sites, cautioning that exposure divergence may not always reflect microclimatic adaptation (Kurze, Bareither, and Metz 2017). Our results for two species and across 15 sites provide perhaps the first direct evidence via fitness differences. They suggest for at least one species, *B. hybridum*, that south exposures have indeed potential to harbor nearby outposts of genotypes that are better adapted to the drier and shorter growing seasons that are expected under climate change in our region (Hochman et al. 2020; Drori et al. 2021). It is furthermore noteworthy that the drier conditions at south exposures result chiefly from higher solar radiation that increases temperatures and evapotranspiration (while the macroclimatic gradient results chiefly from decreasing rainfall), suggesting that south exposure plants could possess additional adaptation to higher temperatures, i.e. to another climate change driver that was unfeasible to incorporate in the present study.

## 5 Conclusions

Our study disentangled the role of three key factors for local adaptation along gradients of increasing aridity: competition, short season length, and drought stress. We showed consistent differences along the macroclimatic gradient for both annual target species. Plant populations from more mesic macroclimates proved better adapted to competition while populations from drier macroclimates were superior under short growing seasons. These results provide direct evidence that local adaptation to a drier macroclimate comes at the cost of lower competitive ability under benign conditions (Grime 1974, 1977). Vice‐versa, mesic populations will suffer reduced fitness when climate change shortens the growing season in our study region (Drori et al. 2021). We further show that annual species adapt to drier climates chiefly by drought escape via faster life‐cycles (Kigel et al. 2011; Kooyers 2015) as drier populations appeared no better adapted to drought stress per se. Intensified drought stress within the growing season due to fewer, more erratic rain events under climate change (Drori et al. 2021) may therefore jeopardize fitness in addition to reduced mean precipitation. These results support modeling studies that locally adapted populations are susceptible to climate change across their entire range (Valladares et al. 2014; DeMarche, Doak, and Morris 2019).

We also showed for the microclimatic gradient that south‐exposed hill‐slopes have some potential as nearby genetic reservoirs for climate change adaptation in our study region, although perhaps mostly in macroclimatic mesic sites. The microclimatic gradient between north and south exposures may thus offer two pathways to reduce the vulnerability of species under climate change. First, the cooler, more mesic north exposures provide microclimatic shelters from increasing aridity and temperatures at relatively short distance, which is notably a benefit that holds also for (arid) sites without microclimatic local adaptation between exposures. Secondly, a nearby reservoir of drier‐adapted genotypes at south slopes may assist the persistence of populations within a site under changing climate. While we caution that the magnitude of both effects in the field needs further investigation, they can win additional time for further (rapid) adaptive evolution within sites (Franks, Sim, and Weis 2007; Metz et al. 2020) and long‐distance migration along the macroclimatic gradient. Including these effects may provide an intriguing, yet thus far unexplored extension to models forecasting the future distributions of locally adapted species (Valladares et al. 2014; DeMarche, Doak, and Morris 2019).

## Author Contributions

**Florian Gade:** data curation (lead), formal analysis (lead), investigation (lead), methodology (supporting), validation (equal), visualization (lead), writing – original draft (equal), writing – review and editing (supporting). **Johannes Metz:** conceptualization (lead), formal analysis (supporting), investigation (supporting), methodology (lead), project administration (lead), supervision (lead), validation (equal), visualization (supporting), writing – original draft (equal), writing – review and editing (lead).

## Conflicts of Interest

The authors declare no conflicts of interest.

## Data Availability

The data supporting our results is publicly available at https://data.goettingen‐research‐online.de via https://doi.org/10.25625/TKY38D.

## Appendix

**TABLE A1:** Results of linear mixed models testing the effects of annual rainfall along the macroclimatic gradient (rain), exposure (north vs. south), season length (long vs. short) and treatment (standard, drought, and competition) on reproductive biomass of two annual species, *Brachypodium hybridum* and *Hedypnois rhagadioloides*. The models included three nested random factors (site/genotype/individual) and were calculated in package *lme4* (Bates et al. 2015). Significance of the fixed effects was tested via type II Wald *F‐*tests in package *car* (Fox and Weisberg 2019) with Kenward–Roger approximated degrees of freedom. Bold indicates significant *p*‐values (*p* < 0.05).

Source of variation	*B. hybridum*			*H. rhagadioloides*		
	df, df	*F*	*p*	df, df	*F*	*p*
Rain	1, 13.3	22.43	**0.0004**	1, 11.2	11.98	**0.00519**
Exposure	1, 415.6	1.3	0.2554	1, 247.0	2.51	0.11454
Season length	1, 1224.1	2754.01	**< 0.0001**	1, 593.2	2457.49	**< 0.0001**
Treatment	2, 822.9	1350.42	**< 0.0001**	2, 434.9	809.19	**< 0.0001**
Rain × exposure	1, 402.3	7.27	**0.0073**	1, 236.1	4.6	**0.03296**
Rain × season length	1, 1222.9	181.35	**< 0.0001**	1, 591.8	49.13	**< 0.0001**
Exposure × season length	1, 1224.2	0.57	0.4486	1, 593.3	0.07	0.79192
Rain × treatment	2, 820.1	35.37	**< 0.0001**	2, 429.9	19.84	**< 0.0001**
Exposure × treatment	2, 823.5	1.99	0.1379	2, 434.7	10.38	**< 0.0001**
Season length × treatment	2, 1224.2	439.4	**< 0.0001**	2, 594.0	349.88	**< 0.0001**
Rain × exposure × season length	1, 1222.6	5.02	**0.0253**	1, 592.1	0.56	0.45611
Rain × exposure × treatment	2, 820.4	1.26	0.2849	2, 429.8	6.14	**0.00234**
Rain × season length × treatment	2, 1222.9	7.29	**0.0007**	2, 592.2	4.35	**0.01335**
Exposure × season length × treatment	2, 1224.3	3.69	**0.0252**	2, 594.0	1.6	0.20265
Rain × exposure × season length × treatment	2, 1222.6	1.39	0.2507	2, 592.3	2.53	0.08071
